# Excision of Superior Maxilla in a Case of Chondroma

**Published:** 1869-08

**Authors:** H. Wardner

**Affiliations:** Cairo, Illinois


ARTICLE XXIX.

### EXCISION OF SUPERIOR MAXILLA IN A CASE OF CHONDROMA.

By H. WARDNER, M.D., of Cairo, Illinois.

Nimrod Hughes, a private soldier of the Seventh Tennessee Cavalry, entered the U. S. General Hospital, at Mound City, Ill., in the fall of 1864, being sent to the rear from Nashville, on account of a tumor, or rather an enlargement, of the left side of the superior maxilla, which prevented him from taking food except in a fluid form. The tumor had been growing for about five years, but had not been of any great inconvenience until within the last year preceding the above date. It involved nearly all of the alveolar process of the affected side, but had not extended to the body of the bone, so as to cause any enlargement. The tumor protruded internally and externally, and somewhat posteriorly on the outer side. It filled about half the cavity of the mouth. The teeth were greatly distorted: one of the molars pointed horizontally across the mouth, and others turned outward against the cheek. The exploring needle was readily passed into it, and proved it to consist of thin laminæ of bone, with an outer substance of sarcomatious or cartilaginous character. His general health was good. His complexion ruddy and free from that peculiar cachectic appearance attending malignant disease. The diagnosis was, therefore, non-malignant chondroma; and the prognosis, under surgical treatment, was favorable. The patient was willing and anxious to have the diseased bone removed.

Accordingly, on the 20th of December, 1864, assisted by Surgeon Hoard, U. S. N., and Act. Assistant-Surgeons Kellogg and Eno, I proceeded with the operation in the following manner (according to Guérin's plan):—

He was placed in a barber's chair and brought under the influence of chloroform.

I then made an incision from the ala of the nose to the commissure of the lips, following the natural furrow in the skin down to the bone. The two flaps thus formed were dissected from the surface of the bone, until the malar process was freely exposed on one side, and the nasal process and nostril on the other. The lower two-thirds of the width of the malar process was then cut with a pair of Liston's forceps, as near the body of the bone as possible; leaving enough of the process intact to preserve the lower border of the orbit. The corresponding incisor was then extracted.

The division of the soft parts of the roof of the mouth, along the suture, from the last molar tooth to the centre of the palatine arch, was next attempted; but, although the patient was affected by the chloroform to the degree of insensibility, we were unable to open his mouth by any reasonable amount of force, so as to introduce the knife with safety; consequently, that part of the operation was deferred.

One blade of the forceps was then introduced into the nostril, the other being in the mouth; the flat or convex side of the blade was pressed against the septum nasi, and the alveolar and palatine processes were divided at one stroke, care being taken not to injure the palate bone. The convex surface of the blades of the forceps were then turned upwards, and one blade introduced horizontally through the nostril into the autrum, and swept around under the floor of the orbit, when, with one cut, the bone was divided from the nostril to the upper extremity of the division made of the malar process. The part being excised was now free, except its attachments to the pterygoid process and the palate bone. It was then seized with a pair of tooth forceps and drawn downwards and forwards with a sudden jerk, when its bony detachment was complete. An attempt was now made to sever the soft parts of the roof of the mouth, in front of the palate bone, with a pair of curved scissors introduced from the side; but as there was not room to use them successfully, the excised bone was drawn forward and toward the opposite side, and the parts divided with a small scalpel, which was passed over the bone. This completed the removal.

The hemorrhage was very slight, no ligature being required.

The flaps were carefully drawn together with silver wire sutures. Narrow strips of adhesive plaster were applied between the sutures, and the whole covered with a coating of collodion. This dressing kept the flaps in perfect coaptation, and union, by "first intention," took place.

A compress of lint dipped in cold water was placed in the cavity left by the removal of the bone, and changed very frequently for two or three days.

The wound made by the removal of the bone healed by granulation, without any complications, in about four weeks, except the opening into the nostril, which was frequently touched with nitrate of silver, for four weeks longer. I saw the patient about five months after the operation, when this opening had contracted to about the size and shape of a common field bean.

The cavity left in the mouth was partially filled by granulation.

His voice had a nasal twang, but he could articulate distinctly.

The deformity of feature was scarcely perceptible. The malar and nasal processes and the floor of the orbit being left intact gave support to the soft parts, so that the outlines of the face were well preserved.

The diseased portion which was removed is now in the Museum of the Chicago Medical College.

Up to the present date (July, 1869), no return of the disease has been observed.

